# Activation, Inhibition, or Something Else: An Exploratory Study on Response Priming Using Moving Dots as Primes in Middle-Aged and Old Adults

**DOI:** 10.1155/2018/7432602

**Published:** 2018-06-19

**Authors:** Christina Bermeitinger, Cathleen Kappes

**Affiliations:** University of Hildesheim, Hildesheim, Germany

## Abstract

Response priming refers to the finding that a prime stimulus preceding a target stimulus influences the response to the following target stimulus. With young subjects, using moving dot stimuli as primes indicated faster responses to compatible targets (i.e., prime and target are associated with the same response) with short stimulus onset asynchronies (SOAs). In contrast, with longer SOAs, participants responded faster to incompatible targets. In the present study, we extended the evidence by comparing middle-aged (50–65 years) and old (66–87 years) adults. With two different motion types, the result found in young participants was replicated in the middle-aged adults. In contrast, old adults showed large positive compatibility effects with the short SOA but neither activation nor inhibition effects with the longer SOA. We discuss our findings in light of several theoretical accounts (i.e., inhibitory deficit, deautomatization, evaluation window account, attention, rapid decay).

## 1. Introduction

There are various kinds of motion surrounding us in our daily life. Perception and discrimination of different kinds of motion represent an important ability, already present in newborns (e.g., [1, 2]). Several research traditions have investigated the special role of motion and moving things in perception and attention (e.g., [3, 4]), and there has been an increased impact of motion research on cognitive theories since common coding approaches to perception and action have been provided (e.g., [5]). In these approaches, it is assumed that perception and action rely on identical cognitive representations. Further, own motion is strongly connected to processes in the motor system as well as to sensory and vestibular feedback. In turn, motor control, sensorimotor functions, motor representations, and general motor performance (for a review, see, e.g., [6]) are subject to age effects, oftentimes studied by functional brain imaging techniques.

Response priming (for a review, see, e.g., [7]) as a behavioral measure investigates the effects of preactivation of motor activations or perceptual/semantic preactivation from a prime event (i.e., a first stimulus) on the processing of a target event (i.e., a second stimulus which has to be categorized). Response priming (please note that, in classical studies on response priming, the same stimuli were used for primes and targets, i.e., in these studies, perceptual priming and response priming cannot be distinguished. However, for the current work, this differentiation is not of central relevance) has mainly been investigated using shape and color stimuli (e.g., [8]). For example, primes and targets are squares and diamonds and the participants' task was to quickly and accurately respond to the shape of the target (e.g., left button press for diamonds and right button press for squares). Reaction times to the target are often reduced when the preceding prime stimulus and the target are associated with the same response (i.e., primes and targets are congruent, consistent, or compatible; e.g., both are squares) compared to targets that are associated with another response (i.e., primes and targets are incongruent, inconsistent, or incompatible; e.g., the prime is a square, the target is a diamond).

Recently, Bermeitinger [9] introduced a variant of response priming in which moving dot rows were used as primes (henceforth “row-of-dots” primes) for static arrow targets to investigate preactivations of directional motions. Bermeitinger [9] found—with young samples—compatibility effects with moving prime stimuli (a row of dots moved leftwards, rightwards, or in a neutral direction (i.e., towards the borders or the center of the screen)) on static arrow targets. The sign of the compatibility effect crucially depended on the stimulus onset asynchrony (SOA) between prime and target. Essentially, with an SOA of 50 ms, no priming was observed. With SOAs of 100 and 150 ms, positive compatibility effects emerged. With SOAs between 250 and 500 ms, negative compatibility effects emerged. This pattern appeared whether the SOA was varied between participants or within participants, independently of prime duration [9, 10], and with forced-choice as well as free-choice tasks [11]. In further experiments utilizing a single moving dot (instead of the row of dots) as a prime, we found the same relative pattern, with positive compatibility effects following shorter SOAs and negative compatibility effects following longer SOAs, but over a different absolute time, we found positive effects up to an SOA of 360 ms and negative effects with SOAs of 800 to 1,200 ms [12].

Although the present research was not concerned with testing theoretical explanations for negative compatibility effects, we shortly summarize the most important explanations of it (for a review, see, e.g., [13]). Schlaghecken, Eimer, and colleagues [14–19] argued that negative priming effects (at least with masked primes) reflect an (self-)inhibition mechanism in low-level motor control. That is, the masked prime initially induces a response tendency corresponding to the action associated with the prime. Thus, positive priming effects are produced. Introducing the mask reduces or eliminates prime visibility. Therefore, early motor activation tendencies are no longer supported by sensory evidence, and positive priming is counteracted by inhibition to prevent premature responding. The results of Bermeitinger [9] can also be explained by this account if one assumes that self-inhibition is released independently of another intervening stimulus (i.e., mask) after some time. The inhibitory motor control always might come into operation when motor activations are classified as unfounded or debilitating. Motion seems to have the capacity to trigger responses rapidly and involuntarily (see also [20], Exp. 4). These response activations resulting from perceived motion have to be inhibited very quickly if they are classified as unfounded. Thus, the general mechanism proposed by Schlaghecken, Eimer, and colleagues could operate in response priming with motion primes, too. There are also alternative accounts which could explain negative compatibility effects (especially with masked primes; e.g., [21–23]; for an attentional account, see [24]).

Interestingly, Schlaghecken and Maylor [25] tested participants in old age (71–83 years) with their subliminal response priming paradigm using static arrows as primes and targets. They found substantial positive compatibility effects with short prime-target SOAs (i.e., 33 ms). However, old participants did not produce significant compatibility effects—neither positive nor negative—with longer SOAs (i.e., between 183 and 483 ms). In contrast, young participants showed clear negative effects with (in their case) the long SOA of 183 ms (there were no significant effects with SOAs of 333 and 483 ms). The authors interpreted their findings of missing negative effects with longer SOAs as evidence that, in old age, self-inhibition at the stage of low-level motor control is impaired (or for some participants at least delayed). According to the inhibitory deficit theory of Hasher and Zacks ([26]; an updated version of the theory is given by Lustig et al. [27]), there are age-related inhibition deficits, mainly associated with decreasing functioning in the frontal lobes. Specific deficits in inhibition are evidenced by several tasks and research methods (for an overview, see, e.g., [27]; for a summary of critics on the inhibitory deficit theory, see also, e.g., [28]). A current point of discussion is whether different inhibition processes (e.g., motor versus sensory, modality-specific, etc.) have to be distinguished and whether these different processes are correlated or not (e.g., [28, 29]). The results of Schlaghecken and Maylor extend the evidence of inhibition deficits to low-level motor control (assumed to be needed for negative compatibility effects with subliminal primes), as low-level motor control is associated with subcortical control structures (e.g., basal ganglia, thalamus, cerebellum).

However, empirical findings, as well as theoretical arguments, are at odds with the initial interpretations of Schlaghecken and Maylor [25]. First, Sumner et al. [30] found clear negative compatibility effects in old age (56–75 years, healthy control group) with SOAs of 150, 200, and 300 ms in manual and saccadic responses (but no, or only slight, positive compatibility effects with an SOA of 500 ms), and Seiss and Praamstra [31, 32] found (reduced) negative compatibility effects in middle-aged adults (51–65 years [31]; approx. 38–64 years [32]). That is, low-level motor control and inhibition still seem to be present in old(er) age. Besides, Schlaghecken, Maylor, and Birak [33, 34] themselves observed negative compatibility effects in old age (63–82 years)—especially when target location and response location (left/right) were incongruent (the authors used a combined Simon and priming procedure; i.e., in incongruent Simon trials, responses are generally slower than in congruent Simon trials) and when individually late responses were analyzed. The authors interpreted their findings and (especially these delayed effects) as evidence for impaired automatic low-level control (i.e., a deautomatization) which is replaced by high-level, automatic control processes.

Verleger [35], however, thought that this is not the most parsimonious interpretation of the data. He relied on the mask-triggered inhibition account of Jaśkowski [36, 37] according to whom the mask induces inhibition (instead of an automatic inhibition according to Schlaghecken and colleagues). Further, Verleger refers to Klauer and Dittrich [38] who presented their “evaluation windows account.” Some key assumptions of this account are as follows: (1) participants evaluate all incoming events across a time window, (2) participants learn to partition the stream of incoming evidence into distinct episodes by use of the stimuli occurring during the experiment, (3) the stimulus immediately preceding the target stimulus (this could be the mask or another intervening stimulus, or with unmasked stimuli the prime itself) acts as a go-signal for opening the “evaluation window”—and additionally can be used as temporal marker to close the window due to the prime and cancel activation induced by the prime [35], (4) decisions about stimuli belonging to one of the given (response) categories are influenced by changes in evidence, and (5) negative compatibility effects will result if the prime falls outside the evaluation window, and positive compatibility effects will result if the prime falls inside the evaluation window. Verleger interpreted the findings by Maylor et al. [33] as evidence for “sluggish temporal structuring in the elderly”—that is, older people might have difficulty using the stimuli to segment the incoming stream of events and appropriately open the evaluation window.

In summary, first, there is an ongoing debate on the mechanisms underlying the negative compatibility effect with static stimuli. Second, it is unclear which account is most appropriate for explaining the pattern of results found with motion primes. Third, findings with older adults in response priming are mixed and also the object of theoretical debates. Fourth, until now, there have been no experiments with middle-aged and older adults using motion primes in response priming.

In the present study, we set out to explore some of the open questions. We used motion primes because they afford the opportunity to investigate responses following the same SOA which have led to positive compatibility effects with one prime type (i.e., single-dot motion type) and negative compatibility effects with another prime type (i.e., row-of-dots motion type) in young participants. Therefore, in the present experiments, the same tasks as in Bermeitinger [9] and Bermeitinger and Wentura [12] were used and we tested middle-aged (50–65 years) and old (66–87 years) adults. The division into age groups is based on the normal retirement age in Germany (i.e., between the 65th and 67th birthday for our sample). Participants' task was simply to respond to static target arrows presented at the center of the screen: Participants pressed the left or right key to a left or right arrow with their left or right index finger, respectively. Before each arrow, participants saw one of two motion type primes. Half of the subjects saw a row of 11 dots in the center of the screen (row-of-dots motion type). These dots seemingly moved rightwards, leftwards, to the center, or towards the borders of the screen. The other half of the subjects saw a single dot moving leftwards, rightwards, or alternately left and right (i.e., neutral condition). The shifting of the dots led to the impression of moving dots. The factor Motion Type (row-of-dots versus single-dot) was varied between participants. Further, the stimulus onset asynchrony (SOA) between the first presentation of the dots and the presentation of the arrow was also varied between participants (SOAs of 147 and 360 ms were utilized as these were the most stable SOAs leading to positive compatibility effects (PCEs) with the single-dot as well as the row-of-dots motion, but to negative compatibility effects (NCEs) with the row-of-dots but PCEs with the single-dot motion type, resp.).

Due to the exploratory character of our study, there are several possible outcomes of the experiment. First of all, we expected a main effect of motion type with larger (positive) compatibility effects with single-dot primes than with row-of-dots primes, comparable to the results with young participants in earlier studies [12]. With single-dot primes, we expected no negative compatibility effects at all, as the SOAs used in the current experiments were too short for finding negative effects with this material [12]. Given that negative compatibility effects occurred with an SOA of 360 ms with the row-of-dots motion type [9], we expected at least reduced negative compatibility effects in this SOA condition for the old participants, if they had specific problems regarding inhibition. In contrast, with the single-dot motion type, old adults should show positive compatibility effects in the longer SOA condition comparable to those of young adults [12] if the effects in this SOA condition are independent of inhibition and/or are independent of any kind of automatic decay of activation. Moreover, for the middle-aged age group, we expected either the same pattern as previously found with young participants or a pattern in between young and older participants (e.g., [31]).

## 2. Method

### 2.1. Participants

Overall, 85 participants were tested. We had to exclude 6 participants due to technical reasons during recording and 7 participants due to being outliers regarding their mean error rates. From the remaining participants, 33 (19 female) were of the middle-aged age group (range: 50–65; *M* = 61.03 years, SD = 3.33) and 39 (21 female) were of the old age group (range: 66–87; *M* = 72.31 years, SD = 5.03) (note that the sample sizes of some Age Group × SOA × Motion Type conditions seem rather small. However, power analyses based on our previous experiments (e.g., [12]) showed that some expected effects are very high (e.g., *d* = 2.37 in the 147 ms SOA condition). Thus, the sample size needed to find an effect in this condition is *n*=4 [39]. Due to the exploratory character of our study, we used the incidental sample that could be recruited via newspaper articles or were guest students. That is, we could not equally assign the age groups to the experimental conditions. Further, we did not measure other cognitive capacities (e.g., general processing capacities or intelligence)). Participants had self-reported normal or corrected-to-normal vision (although all participants reported normal or corrected-to-normal vision, it might be that some older adults did not wear the appropriate glasses for the given task. However, the data speak against an interpretation based on vision problems because reactions times in the SOA 147 ms condition were highly comparable between age groups, which renders the explanation that the older participants needed more time to identify the stimuli improbable) and were right-handed, excepting one who was left-handed and four who were without any dominance. Participants were recruited via newspaper announcements or they were guest students at the University of Hildesheim and could be contacted by email or phone. They received 10 EUR for their participation.

### 2.2. Design

A 3 (Motion Direction: rightwards, leftwards, and neutral) × 2 (Arrow Direction: leftwards and rightwards) × 2 (SOA: 147 versus 360 ms) × 2 (Motion Type: single-dot and row-of-dots) × 2 (Age Group: middle-aged and old) design was used. The factors Motion Direction and Arrow Direction were varied within participants, the factors SOA and Motion Type were varied between participants, and the factor Age Group was quasiexperimental. In the tradition of priming experiments, we focused on the compatible (dots moved rightwards and the arrow pointed to the right; dots moved leftwards and the arrow pointed to the left) and incompatible (dots moved rightwards and the arrow pointed to the left; dots moved leftwards and the arrow pointed to the right) conditions. The compatibility effect was computed as the target response time difference between incompatible and compatible trials.

## 3. Material

**Figure 1:**
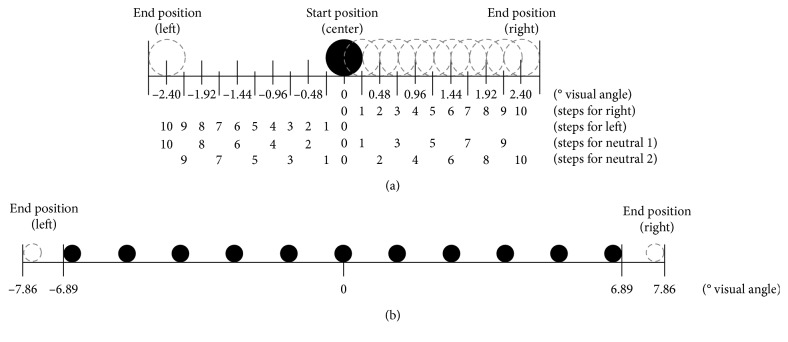
Prime stimuli used in the experiment. Black dots represent the original position of each prime at the center of the screen. Gray circles represent motion positions of the dot(s). For the case of a right moving single dot, all possible dot positions are depicted. The last gray circle on the left or right represents the end position of the motion for left or right movements. Each step lasted one refresh cycle (i.e., 13.33 ms). Note that the scale for the single-dot motion type and the scale for the row-of-dots motion type differ. (a) Single-dot motion primes and (b) row-of-dots motion primes.

All stimuli were presented in black on a white background (see Figure 1 for the primes).

For the row-of-dots motion type, the same material as in Bermeitinger ([9]; see also [12], Exp. 2) was used. Two arrows were used as target stimuli, one pointing to the left and one to the right, and the arrows were approximately 3.34° visual angle (3.5 cm) in length and 0.96° visual angle (1.0 cm) in height. The primes were rows of 10.5, 11, or 11.5 dots; each dot was approximately 0.38° visual angle (0.4 cm) in diameter. The whole row measured approximately 13.78° visual angle (14.5 cm) in length; the distance from one dot to the next dot was approximately 0.96° visual angle (1.0 cm). The prime event started with the presentation of the row at the center of the screen. To instantiate the movement, the dots were shifted from their original position in steps of 0.16° visual angle (0.17 cm) leftwards or rightwards. After six steps, a dot had reached the original position of its neighboring dot and the movement started again from the screen's center (original row position). For each prime event, 11 frames were presented; that is, there were 10 movement steps of the dots. The phenomenal experience is that of a rectangular window that allows a restricted view on an endless chain of moving dots. For the compatible and incompatible conditions, the dots (i.e., the whole row) were moved rightwards or leftwards. For the neutral conditions, the dots were either moved outwards (i.e., the 5.5 left dots of the row moved leftwards and the 5.5 right dots of the row moved rightwards, meaning that the central dot was split into two semicircles that drifted apart) or inwards (i.e., the 5.5 left dots of the row moved rightwards and the 5.5 left dots of the row moved leftwards, meaning that the central dot was split into two semicircles that progressively superimpose; for more details, see [9]).

For the single-dot motion type, the same material as in Bermeitinger and Wentura [12] was used. The target arrows were approximately 2.63° visual angle (2.75 cm) in length and 0.96° visual angle (1.0 cm) in height. A single dot (approximately 0.48° visual angle (0.5 cm) in diameter) was used as the prime. To instantiate the left or right movements, the dot was shifted from its original position in 0.24° visual angle (0.25 cm) steps leftwards or rightwards. In the neutral condition, the dot moved one step to the right (neutral 1) or to the left (neutral 2) and then to the opposite side (i.e., to the left or to the right, resp.); left and right steps alternated successively. For each prime event, 11 frames (each with a single dot) were presented, that is, 10 movement steps of the dot. The end positions of the dots were 2.63° visual angle (2.75 cm) to the left of the original position (i.e., the center) for left movements (and the neutral 1 condition, which started with a right movement) and 2.63° visual angle (2.75 cm) to the right of the original position for right movements (and the neutral 2 condition, which started with a left movement) (please note that these neutral motions are not really neutral; probably, both responses (left and right) are activated, rather than neither. However, a really neutral motion with a single dot seems either impossible or associated with other problems by changing the perceptual properties of the display (e.g., keeping the dot stationary would eliminate not only directional information, but also motion in general; adding a second dot would change the luminance and the number of moving elements). In general, “neutral” conditions are most often not really neutral in each sense. Therefore, the comparison with neutral conditions is usually coupled with some problems).

### 3.1. Procedure

Participants were individually tested in sound-attenuated chambers. The experiment was run using E-Prime software (version 1.3) with standard PCs and 17″ CRT monitors with a refresh rate of 75 Hz. Stimulus presentation was synchronized with the vertical retrace signal of the monitor. Viewing distance was about 60 cm. Instructions were given on the CRT screen. Participants were requested to quickly and accurately categorize each arrow with regard to its direction (by pressing the right/left key with their right/left index finger for right/left arrows, resp.; response keys were the 3 and 1 key on the numeric pad on which a right or left arrow was pasted, resp.). They were told that the dots are irrelevant to their task. The sequence of each trial in the row-of-dots condition was as follows: first a fixation stimulus (+) appeared at the center of the screen for 1,000 ms. It was followed by the first row of dots, which was presented for one refresh cycle (i.e., approximately 13 ms). Then, the next 10 rows of dots were presented for the next 10 refresh cycles, respectively (i.e., resulting in a prime duration of overall 147 ms) (note, after six refresh cycles, the row sequence started again from the original row). The prime event was followed immediately by the target in the 147 ms SOA condition or by a blank screen of 213 ms in the 360 ms SOA condition, which was then followed by the target. The target remained on the screen until a response was given. The intertrial interval was 400 ms.

In the single-dot condition, the same trial procedure was used as for the row-of-dots condition with the following exceptions. First, the dot moved constantly leftwards or rightwards (instead of repeating from the original position after six cycles) in the compatible and incompatible conditions. Second, in the neutral condition, the dot first moved one step to the right (neutral 1) or to the left (neutral 2) and then to the opposite side (i.e., to the left or to the right, resp.). Left and right steps alternated successively.

Each participant worked through four blocks with 36 trials each. Each block consisted of 12 compatible trials (6 with dots moving rightwards and leftwards, resp.), 12 incompatible trials (6 with dots moving rightwards and leftwards, resp.), and 12 neutral trials (6 with dots moving outwards and inwards, resp.); half of the trials had right arrow targets, and the other half had left arrow targets. There was a short pause after each block. Before the first experimental block, there was a practice phase with 12 trials.

The whole experiment (including instructions, breaks, experimental trials) took about 7 to 10 minutes for each person. Before the response priming experiment, all participants took part in a completely unrelated other response time study with words and a lexical decision task.

## 4. Results

Mean reaction times (RTs) were derived from correct responses. Outlying RTs that were 1.5 interquartile ranges above the third quartile with respect to the individual distribution [40], were above 1500 ms, or were below 200 ms were discarded. Due to these outlier and error criteria, 3.4% of all trials had to be excluded (0.8% due to errors). As error rates were extremely low, we did not analyze them further.

**Figure 2:**
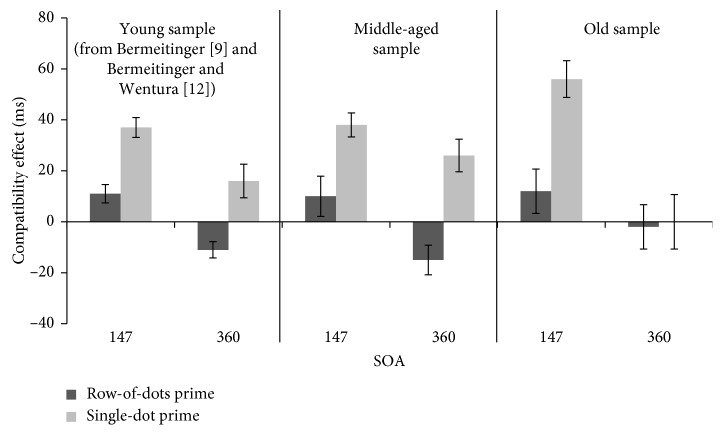
Mean compatibility effects (mean RT incompatible–mean RT compatible) from the 147 and 360 ms SOA condition, separately depicted for the middle-aged and old samples. For reasons of comparison, we added the corresponding priming effects from young samples from experiments published in previous papers with the same conditions as in the current experiment (for the single-dot condition from [12], Exp. 1; for the row-of-dots condition from [9], Exp. 3); error bars represent the standard error of the mean.

Mean RTs and mean compatibility effects for each condition are shown in Table 1 and Figure 2.

Mean reaction times were subjected to a mixed analysis of variance (ANOVA) with the within-participants factors Motion Direction (rightwards versus leftwards) and Arrow Direction (leftwards versus rightwards) and the between-participants factors SOA (147 ms versus 360 ms), Motion Type (single-dot versus row-of-dots), and Age Group (middle-aged, old). Results that are not subsequently reported were not significant (*p* > 0.10, two-tailed) (this even holds true for the main effect “Age Group” and the interaction of “Age Group” and SOA, both ps > 0.10).

The main effect Arrow Direction was significant, *F*(1, 64) = 4.88, *p*=0.03, *η*_p_^2^ = 0.07, indicating slightly faster RTs with the right hand (i.e., arrow pointing to the right) than with the left hand (i.e., arrow pointing to the left). More interestingly, the interaction of Motion Direction and Arrow Direction was significant, *F*(1, 64) = 26.31, *p* < 0.001, *η*_p_^2^ = 0.29, indicating overall a positive compatibility effect. This effect was qualified by significant interactions of Motion Direction, Arrow Direction, and “SOA”, *F*(1, 64) = 19.86, *p* < 0.001, *η*_p_^2^ = 0.24; of Motion Direction, Arrow Direction, and Motion Type, *F*(1, 64) = 22.93, *p* < 0.001, *η*_p_^2^ = 0.26; and, most interestingly, of Motion Direction, Arrow Direction, “SOA,” Motion Type, and “Age Group,” *F*(1, 64) = 5.23, *p*=0.025, *η*_p_^2^ = 0.08 (these effects were also (at least marginally) significant if we included the neutral condition into analysis). Therefore, we further conducted mixed ANOVAs with the factors Arrow Direction, Motion Direction (both within-subjects), Motion Type, and Age Group (both between-subjects) separately for each SOA condition.

### 4.1. 147 ms SOA Condition

For the 147 ms SOA condition, we found a significant Arrow Direction × Motion Direction interaction, *F*(1, 31) = 52.32, *p* < 0.001, *η*_p_^2^ = 0.63, and a significant interaction of Arrow Direction, Motion Direction, and Motion Type, *F*(1, 31) = 20.25, *p* < 0.001, *η*_p_^2^ = 0.40: there were stronger positive compatibility effects with single-dot primes (*M* = 49 ms, SE = 5.2, *t*(15) = 9.45, *p* < 0.001) than with row-of-dots primes (*M* = 11 ms, SE = 5.8, *t*(18) = 1.91, *p*=0.07), independent of age group (compatibility effects in both age groups were of comparable size).

(Positive) benefits are defined as the facilitation in compatible trials compared to neutral trials, and (positive) costs are defined as the slowing in incompatible trials compared to neutral trials (i.e., negative benefits = faster neutral than compatible trials; negative costs = faster incompatible than neutral). Compatibility effects (incompatible versus compatible trials) might be made up of benefits and costs. In fact, the positive compatibility effect in short SOAs splits into significant benefits, *F*(1, 31) = 18.52, *p* < 0.001, *η*_p_^2^ = 0.374–*F* < 1 for the moderation by age and *F*(1, 31) = 17.23, *p* < 0.001, *η*_p_^2^ = 0.357 for the moderation by motion type, with benefits with single-dot primes and no benefits with row-of-dots primes, and significant costs, *F*(1, 31) = 39.07, *p* < 0.001, *η*_p_^2^ = 0.448–*F* < 1 for the moderation by age and *F*(1, 31) = 4.24, *p*=0.04, *η*_p_^2^ = 0.125 for the moderation by motion type, with larger costs with single-dot primes than with row-of-dots primes. Compatibility effects at the short SOA thus were composed of facilitation after compatible row-of-dots primes and deceleration after both incompatible motion type primes.

A further analysis evaluated the correlation between mean reaction time and the size of the compatibility effect in the 147 ms SOA condition, yielding a trend towards a negative correlation (*r*(*n*=35)=−0.31, *p*=0.07). That is, faster participants showed a slightly larger positive compatibility effect (note that age and mean RT are not correlated, *r*(*n*=35)=0.09, *p*=0.61).

### 4.2. 360 ms SOA Condition

In the 360 ms SOA condition, we found a significant main effect of Arrow Direction, *F*(1, 33) = 4.79, *p*=0.04, *η*_p_^2^ = 0.13, and a significant main effect of “Age Group,” *F*(1, 33) = 6.55, *p*=0.02, *η*_p_^2^ = 0.17: participants of the old age group responded more slowly than participants of the middle-aged age group. Further, we found significant interactions of Arrow Direction, Motion Direction, and Motion Type, *F*(1, 33) = 5.84, *p*=0.02, *η*_p_^2^ = 0.15, as well as of Arrow Direction, Motion Direction, Motion Type, and “Age Group,” *F*(1, 33) = 4.60, *p*=0.04, *η*_p_^2^ = 0.12: we found no compatibility effect in the old age group (neither with single dot, *M* = 0 ms, SE = 10.7, *t*(7) = 0, *p*=1.0, nor with row of dots, *M* = −2 ms, SE = 8.7, *t*(10) = 0.28, *p*=0.79). In contrast, in the middle-aged age group, we found the same pattern as found in previous experiments with young participants in the 360 ms SOA condition (see [9, 12]): there was a positive compatibility effect with single-dot primes (*M* = 26 ms, SE = 6.4, *t*(12) = 4.06, *p*=0.002) and a negative compatibility effect with row-of-dots primes (*M* = −15 ms, SE = 5.8, *t*(4) = 2.64, *p*=0.058) (to further substantiate these findings, we additionally conducted regression analyses in which age was treated as continuous variable. Compatibility effects of each condition (SOA × Motion Type) were linearly regressed on age. In the 147 ms SOA condition with single-dot primes, there was a trend towards a positive association between age and the compatibility effect, *β* = 0.46, *t*(14) = 1.92, *p*=0.076: with each year, the compatibility effect increases by 1.56. In the 360 ms SOA condition with single-dot primes, there was a negative association between age and the compatibility effect, *β* = −0.44, *t*(19) = −2.13, *p*=0.046: with each year, the compatibility effect decreases by 1.44. In the 147 ms SOA condition with row-of-dots primes, there was no association between age and the compatibility effect, *β* = 0.11, *t*(17) = −0.30, *p*=0.77. In the 360 ms SOA condition with row-of-dots primes, there was a trend towards a positive association between age and the compatibility effect, *β* = 0.45, *t*(14) = 1.89, *p*=0.08: with each year, the negative compatibility effect decreases by 1.65).

With regard to benefits and costs at the longer SOA, we analyzed the effects separately for age groups. The benefits and costs in middle-aged subjects were moderated by motion type, *F*(1, 16) = 9.09, *p*=0.008, *η*_p_^2^ = 0.362 for benefits and *F*(1, 16) = 8.59, *p*=0.01, *η*_p_^2^ = 0.349 for costs: there were positive benefits and positive costs with single-dot primes and negative benefits as well as negative costs with row-of-dots primes. Thus, the positive compatibility effect with single-dot primes at the longer SOA for middle-aged subjects is composed of facilitation after compatible primes and deceleration after incompatible primes, and the negative compatibility effect with row-of-dots primes is composed of deceleration after compatible primes and facilitation after incompatible primes. The benefits and costs in old subjects were moderated (at least by trend) by motion type, *F*(1, 17) = 3.15, *p*=0.09, *η*_p_^2^ = 0.156 for benefits and *F*(1, 17) = 3.14, *p*=0.09, *η*_p_^2^ = 0.156 for costs: there were negative benefits and positive costs with single-dot primes and positive benefits as well as negative costs with row-of-dots primes.

### 4.3. Correlation between Mean Reaction Time and Compatibility Effect

A further analysis evaluated the correlation between mean reaction time and the size of the compatibility effect, yielding no correlation (*r*(*n*=21)=−0.16, *p*=0.48) in the single-dot condition and a trend towards a positive correlation (*r*(*n*=16)=0.44, *p*=0.09) in the row-of-dots condition. That is, in this condition, faster participants showed a larger negative compatibility effect (note that age and mean RT are correlated in the 360 ms conditions, *r*(*n*=21)=0.51, *p*=0.02, and *r*(*n*=16)=0.63, *p*=0.01, for the single-dot and row-of-dots condition, resp.).

## 5. Discussion

In the present study, we conducted a response priming task using moving dots as primes for static arrow targets. We tested two different motion types (single-dot primes and row-of-dots primes) at SOAs of 147 ms and 360 ms with middle-aged (50–65 years) and old (66–87 years) participants. In summary, for middle-aged adults, we found the same pattern of results as with younger participants of previous studies: there were positive compatibility effects with single-dot primes independent of SOA, positive compatibility effects with row-of-dots primes in the short SOA condition, and negative compatibility effects with row-of-dots primes in the longer SOA condition. Further, as expected, in the short SOA condition, middle-aged and old participants showed the same pattern of results with positive effects in both motion type conditions and the age groups showed impressively similar reaction times in the motion type conditions (Table 1). Importantly, there was a difference between middle-aged and old participants in the longer SOA condition: old participants showed no compatibility effects, neither a positive effect with single-dot primes nor a negative effect with row-of-dots primes. That is, separate things seem to happen after 147 ms in middle-aged and old adults. In the following, we discuss these main findings of our experiment.

First, as expected [12], we found overall larger compatibility effects with the single-dot motion type than with the row-of-dots motion type. This could be further evidenced that different motion types trigger initial activation processes differently. The single-dot primes seem to release larger and/or more directed and focused activation than row-of-dots primes. When looking at benefits and costs, compatibility effects with single-dot primes are composed of facilitation and deceleration. In contrast, (positive) compatibility effects with row-of-dots primes solely are based on deceleration. There is an ongoing debate on the relationship between activation and inhibition processes. Generally, it is assumed that inhibition is released only when a high level of internal activation is reached (e.g., [41]). However, in our case, activation after row-of-dots primes might be more blurred—as might be the case with masked primes, too. Thus, the requirement of inhibition might be stronger after row-of-dots primes. A further explanation might be that single dot and row of dots are differently suitable as triggers for opening the evaluation window [38]. According to this account, negative compatibility effects will result if the prime falls outside the evaluation window and positive compatibility effects will result if the prime falls inside the evaluation window. In turn, row-of-dots primes might lead to a later opening of the evaluation window which does not include the prime, resulting in negative compatibility effects. In contrast, single-dot primes might act as a signal to open the evaluation window with their start (or vice versa, they might be less distinguishable from the entire stream of incoming events and not act as a signal to open the evaluation window with their *end*), resulting in positive compatibility effects. In either case (impaired inhibition and evaluation windows account), our results again showed that the role of a mask (e.g., Jaśkowski, 2007, 2008) [35] is not decisive—as we had no mask at all.

Second, we found exactly the same pattern in the middle-aged age group as with young participants in previous studies [9, 12]: there were positive compatibility effects with both motion types in the short SOA condition (as expected, larger effects with the single-dot motion type than the row-of-dots motion type) and positive compatibility effects in the longer SOA condition with the single-dot motion type, but negative effects with the row-of-dots motion type. This pattern can be seen as evidence that middle-aged participants have no obvious relevant impairments of activation, inhibition, attention, and/or decay processes, as compared to young participants. Our finding is somewhat different to that of Seiss and Praamstra [31] who found only reduced negative compatibility effects with middle-aged participants. Probably, motion has a generally higher salience than static symbols. Additionally, directional motion is closely related to spatial attention (e.g., [42]), again also due to the fact that motion is a nonsymbolic prime which might trigger an own motion directly, whereas static stimuli (e.g., shapes, colors, words) represent symbolic and abstract information which has to be transformed from the symbol to task-specific requirements in a first step (we thank Friederike Schlaghecken for suggesting this point). Thus, activation and following inhibition can be released faster than with static stimuli.

Third, in the short SOA condition, we found comparable positive compatibility effects in middle-aged and old participants (for both motion types) with even slightly larger compatibility effects in the old than the middle-aged age group with single-dot primes. That is, participants of both age groups preactivated the response tendency associated with the prime (at least after single-dot primes), resulting in faster responses to compatible than incompatible targets. This finding perfectly matches previous findings showing sustained or even enhanced activation [25, 27]. In neuroimaging studies, larger and/or more distributed activation in older compared to younger persons has oftentimes been found (for a review, see, e.g., [43]). There is ongoing debate as to whether the activation patterns in old age reflect compensation or inappropriate processing (for reviews, see, e.g., [44, 45]). Overall, we see the pronounced positive compatibility effects in the short SOA condition as evidence that there are no general activation deficits, especially not in the old age group. This is comparable to other findings in which there was no age-related decrement in initial activation (e.g., [25]).

Fourth and most interesting, there are differences between the age groups in the longer SOA conditions—here, middle-aged participants showed positive compatibility effects with single-dot primes and negative compatibility effects with row-of-dots primes. This mirrors the effects for young adults. In contrast, we found no compatibility effects in old participants in the longer SOA conditions. First, the lack of negative compatibility effects with row-of-dots primes matches expectations based on other findings and can be explained with reduced inhibition and impaired low-level motor control (e.g., [25]), as well as with the evaluation windows account [38] and the assumption of a more sluggish temporal processing in old age [35]. According to the deautomatization account by Maylor et al. [33], we would have expected larger negative compatibility effects with longer response times in the longer SOA condition (especially for row-of-dots primes). However, our correlation analysis showed—by trend—the opposite pattern: shorter response times are associated with larger negative compatibility effects (but note that age and response times are also correlated here). Second, according to previous findings and theoretical ideas, we should have found positive compatibility effects with single-dot primes (comparable to young and middle-aged participants), as activation processes are not assumed to be reduced in older age (e.g., [27]). The lack of (positive) compatibility effects with single-dot primes in the longer SOA is thus surprising.

How can this be explained? We again may refer to the theories already mentioned above. Perhaps, with larger initial activations in old people, the requirement of faster/earlier inhibition is given. Thus, the finding of no compatibility effect might reflect an intermediate state and lead subsequently to a negative compatibility effect with longer SOAs which might be an interesting question for future research. Note that, for young participants and single-dot primes, negative compatibility effects may only occur with rather long SOAs (above 800 ms). Alternatively, the deautomatization of low-level control could lead to controlled inhibition with the same time course independently of material and initial activation resulting in the same null effect with row-of-dots and single-dot primes. Last, but not least, we propose a further explanation whereby our results are evidence that activation processes change with age (in contrast to the inhibitory deficit theory; e.g., [27]).

Accordingly, a plausible alternative assumption is that, in older adulthood, activation processes are also altered, or more specifically, there might be a lack of (preceding) sustained activation in longer SOAs. Thus, inhibition is not initiated due to this lack of sustained activation. As found in other studies (e.g., [27, 46]), there is larger initial activation in older age, often interpreted as reflecting compensatory processes. Rapid decay of activation, in turn, could be caused by such compensatory processes. Such processes might limit the capacity to sustain the initial activation. Thus, no positive compatibility effects occur with the longer SOA of 360 ms after an initially large positive compatibility effect with the short SOA of 147 ms in the old age group. However, the results of Schlaghecken and Maylor [25] seem to stand at odds with this interpretation as they found positive priming effects (i.e., activation) in older adults, but not negative priming effects (i.e., inhibition). However, they tested positive and negative compatibility with different SOAs, of course (i.e., 33 ms for PCEs and 183 ms for NCEs, see above). Due to differences in timing caused by different SOAs and resulting in different time distances between prime presentation/initial activation release and target presentation/response execution, it is unclear whether the lack of compatibility effects in old age is caused by impaired automatic inhibition or whether there is a more rapid activation decay than in younger persons (please note that this explanation will not conflict with the deautomatization account, e.g., [33]). That is, it could be that, in old age, (1) activation decays rapidly without the necessity to initiate inhibition processes, or (2) no initiation of automatic inhibition processes is actually possible. Both explanations would result in a lack of negative compatibility effects with longer SOAs, even after a high level of initial activation (expressed in positive priming effects with shorter SOAs). With our paradigm, it was possible to compare conditions in which positive and negative priming effects occur at the same SOA of 360 ms (single-dot primes: positive priming effects; row-of-dots primes: negative priming effects) in young age. Thus, the results with old participants can also be explained by a rapid decay of initial activation, which removed the need for the release of inhibitory processes. Thus, we propose the interpretation that the lack of compatibility effects can be seen as evidence for a rapid decay of initial activation.

Additionally, we would like to propose a further argument for our point that the lack of negative compatibility effects in old age is not (fully) caused by impaired automatic inhibition. In contrast to some response priming experiments, we included a neutral condition in which the prime is associated with neither the left nor the right response; thus, no specific directional preactivation is possible. With the row-of-dots motion type in the short SOA condition, we found no differences between compatible and the neutral conditions, neither in the middle-aged age group, nor in the old age group, nor in comparable experiments with young age groups ([9], Exp. 1, Exp. 3). In contrast, the positive compatibility effects result from response time differences between the incompatible and the neutral condition (i.e., costs)—we found slower responses in incompatible than neutral trials. That is, some kind of inhibition process was released for incompatible targets, slowed the responses to them, and caused the overall positive compatibility effect. As there is no difference between age groups, this kind of inhibition also occurs in the old age group. We take this as evidence that inhibition is not generally impaired (in response priming tasks) in old age. Further, with single-dot primes, the missing compatibility effect in old participants is the result of decelerations after both compatible and incompatible primes (compared to neutral). That is, each kind of directional activation seems to be downregulated or decayed. With row-of-dots primes, the compatibility effect is also cancelled out by equally high facilitation after compatible and incompatible primes (compared to neutral).

Finally, as already pointed out by Bermeitinger [9], our pattern of results also matches results from another background, that is, from attention research (see also [24]). This is especially relevant as we used somewhat unusual targets that were slightly larger than the region of foveal vision. Research on spatial attention shows that when responding to a target stimulus, people are typically slower when it appears at least approximately 200 ms later at the same location as a previous event (up to 200 ms, benefits often are found). The phenomenon is often called inhibition of return (IOR; for a review, see, e.g., [47]). Castel et al. [46] investigated the time course of IOR in young and old participants. The results showed larger initial facilitation at shorter SOAs in older than in younger participants—comparable to our findings here—and 300 ms delayed IOR effects in older than in younger participants—comparable to the findings of, for example, Maylor et al. [33]. Castel et al. did not try to explain their results, except for the comment that there might be changes in the temporal dynamics of inhibition with age. Overall, it is open to future research how far IOR and priming research could and should be considered together.

To conclude, the patterns (at least parts of them) found in our study can be explained with recourse to several theories (i.e., impaired low-level motor control, evaluation windows account, attention theories) and even as evidence for a rapid decay of initial activation and not (or not exclusively) impaired inhibition in older age. Our study had the advantage that we could measure positive and negative compatibility effects at the same SOA by means of two different motion types as primes. Different elapsed time between prime and target as well as explanations relying on some kind of mask can be excluded as explanations for differences in compatibility effects and our results in general. Thus, the specific material and task are suitable for future research to study further differences between age groups and activation, inhibition, timing, and/or decay processes. Future research should also test some of the explanations in direct comparison with each other which was not the in the scope of the current (exploratory) study.

## Figures and Tables

**Table 1:** Mean response times (in ms; SD in parentheses) of the compatible, the incompatible, and the neutral conditions as well as the compatibility effects (incompatible–compatible; in ms; SE in parentheses; discrepancies due to rounding differences) for each SOA (147 versus 360 ms) × Motion Type (single-dot versus row-of-dots) × Age Group (middle-aged, old) condition. Additionally, the effects of young participants (MED_age_ = 22 years, ranging from 18 to 47) in the single-dot ([12], Exp. 1, 147 and 360 ms SOA conditions) and row-of-dots ([9], Exp. 3) conditions from experiments published in previous papers with the same conditions as in the current experiment are presented for a direct comparison.

	Young (results from previous papers)		Middle aged								Old age							
	Single-dot motion type	Row-of-dots motion type	Single-dot motion type				Row-of-dots motion type				Single-dot motion type				Row-of-dots motion type			
	Effect	Effect	Compatible	Incompatible	Neutral	Effect	Compatible	Incompatible	Neutral	Effect	Compatible	Incompatible	Neutral	Effect	Compatible	Incompatible	Neutral	Effect
147 ms SOA	37 (3.9)	11 (3.6)	429 (66.3)	467 (73.3)	452 (63.3)	38 (4.7)	466 (53.2)	476 (37.4)	466 (38.7)	10 (7.9)	429 (64.8)	485 (47.6)	457 (53.3)	56 (7.2)	464 (63.0)	475 (59.4)	464 (66.5)	12 (8.7)
360 ms SOA	16 (6.6)	−11 (3.2)	406 (31.7)	431 (30.9)	421 (32.6)	26 (6.4)	440 (34.6)	424 (27.9)	434 (27.5)	−15 (5.8)	456 (30.9)	456 (39.0)	447 (33.2)	0 (10.7)	465 (47.2)	463 (61.5)	473 (61.4)	−2 (8.7)

## Supplementary Materials

Supplementary Materials As supplementary material, we provide the original raw data for all participants. (i) Trials 1–12: practice trials; (ii) Sex = sex (maennlich = male; weiblich = female); (iii) arrow direction: Target = links.bmp = leftwards; Target = rechts.bmp = rightwards; (iv) prime direction: Procedure = rechts = rightwards, Procedure = links = leftwards, Procedure = neutralInnen = neutral inwards, Procedure = neutralAussen = neutral outwards; (v) SOA: ExperimentName ≫ _150 versus _360; (vi) motion type: ExperimentName ≫ row-of-dots = S1 and S2 and single-dot S_P1 and S_P2; (vii) reaction time in each trial: Target.RT; and (viii) correctness of response in each trial: Target.ACC.
